# Automated image registration of cerebral digital subtraction angiography

**DOI:** 10.1007/s11548-023-02999-8

**Published:** 2023-07-17

**Authors:** Vincent J. W. Hellebrekers, Theo van Walsum, Ihor Smal, Sandra A. P. Cornelissen, Wim H. van Zwam, Aad van der Lugt, Matthijs van der Sluijs, Ruisheng Su

**Affiliations:** 1 Erasmus MC, University Medical Center, Rotterdam, The Netherlands; 2 Maastricht University Medical Center, Maastricht, The Netherlands

**Keywords:** Digital subtraction angiography, Ischemic stroke, Endovascular thrombectomy, Image registration

## Abstract

**Purpose:**

Our aim is to automatically align digital subtraction angiography (DSA) series, recorded before and after endovascular thrombectomy. Such alignment may enable quantification of procedural success.

**Methods:**

Firstly, we examine the inherent limitations for image registration, caused by the projective characteristics of DSA imaging, in a representative set of image pairs from thrombectomy procedures. Secondly, we develop and assess various image registration methods (SIFT, ORB). We assess these methods using manually annotated point correspondences for thrombectomy image pairs.

**Results:**

Linear transformations that account for scale differences are effective in aligning DSA sequences. Two anatomical landmarks can be reliably identified for registration using a U-net. Point-based registration using SIFT and ORB proves to be most effective for DSA registration and are applicable to recordings for all patient sub-types. Image-based techniques are less effective and did not refine the results of the best point-based registration method.

**Conclusion:**

We developed and assessed an automated image registration approach for cerebral DSA sequences, recorded before and after endovascular thrombectomy. Accurate results were obtained for approximately 85% of our image pairs.

**Supplementary Information:**

The online version contains supplementary material available at 10.1007/s11548-023-02999-8.

## Introduction

Ischemic stroke is the most common type of stroke (71%), a leading cause of disability and death [1]. Endovascular thrombectomy (EVT) aims to restore blood flow by mechanical removal of the thrombus. Intermittently, digital subtraction angiography (DSA) is used to visualize and study [2] the vessels.

A quantitative comparison of the vessels (or perfusion) before and after the intervention may lead to a better understanding of the result of the intervention and may also permit prediction of outcome [3, 4]. Such a comparison of DSA series is currently hampered by the lack of an accurate spatial alignment [5] for series obtained before and after the treatment.

Automating such alignment is challenging, as there may be new arteries visualized after a (partially) successful thrombus removal. Additionally, spatial correspondence likely requires a nonlinear deformation, even for subsequent frames, as is indicated by early work [6] on DSA image processing. Finally, the orientation of the imaging setup, with respect to the patient, can vary significantly during a procedure, as the ischemic stroke patient will move during the procedure. Additionally, the radiologist changes the orientation intermittently for anterior–posterior (AP) or lateral views.

**Fig. 1:**
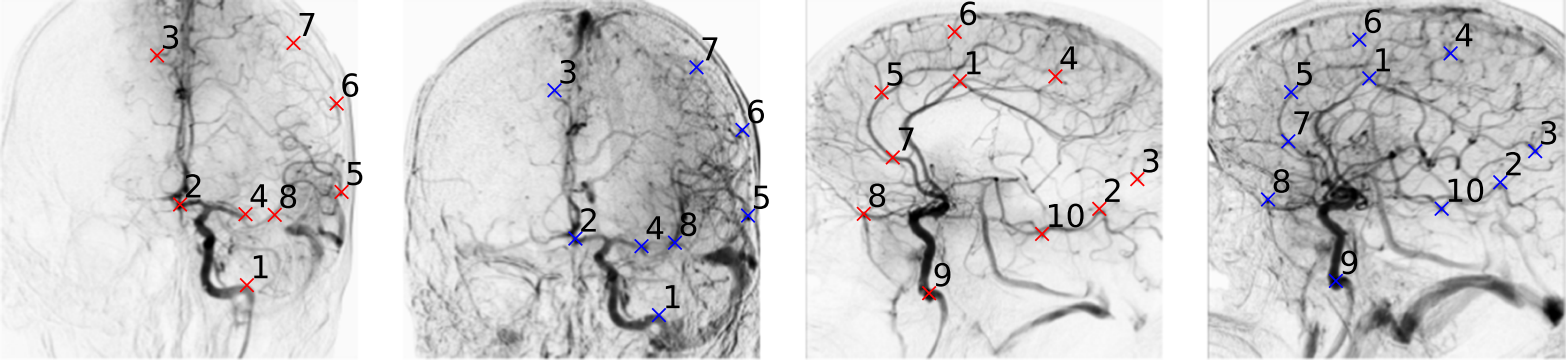
DSA images of an ischemic stroke patient, including annotated point-correspondences for pre-/post-EVT (red/blue) minIPs for AP and lateral views (left/right)

**Fig. 2:**
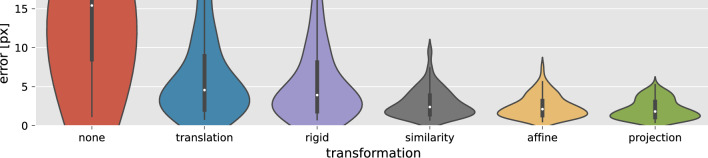
Average residual error distributions of optimized transformations for AP image pairs

In this work, we aim to develop and assess an image registration strategy on a large set of images using a quantitative metric. We will first investigate which type of transformation is effective in aligning different DSA series. Subsequently, traditional registration methods and a deep learning method are adapted and assessed for automated alignment.

## Methods

The effects of patient movement and differences in C-arm orientation, inherently present in DSA data, may require additional transformation complexity for effective alignment. Ultimately, it is not apparent what transformation type is suitable to model the projection of 3D motion. We therefore empirically investigate what transformation type is suited for spatial alignment by fitting different 2D transformations to manually annotated point correspondences.

Subsequently, we assess automatic registration techniques. We first develop a neural network to identify two cerebral artery landmarks, which will provide point correspondences for all DSA sequences. For more accurate alignment of sequences pre- and post-EVT images of the same patient, point correspondences from traditional methods, SIFT (scale-invariant feature transform) [7] and ORB (Oriented FAST and Rotated BRIEF) [8], are used.

**Fig. 3:**
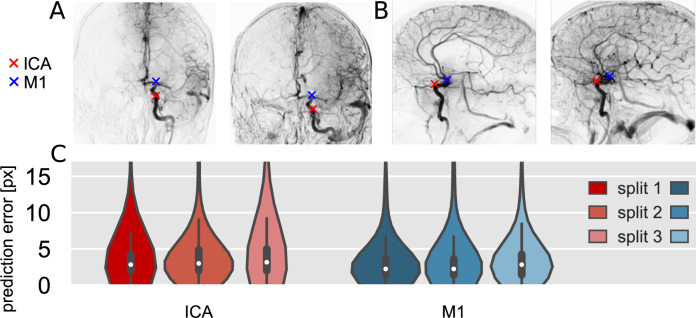
ICA and M1 landmark predictions for **A** AP and **B** lateral MinIPs. **C** AP landmark prediction error distributions for the three data splits for the best-performing configuration

The neural network uses the U-net architecture [9] to compute the probability distributions of the location of the two landmarks (see Fig. 3). The final sigmoid activation function enforces the lower and upper bounds of the probability values. At inference, the landmark positions are determined by the highest probability (argmax) or expectation (centre of mass). Kullback–Leibler (KL) divergence [10] and Jensen–Shannon (JS) divergence [11] are used as loss functions w.r.t Gaussian distributions centred on manual annotations.

## Data

In this work, we use imaging data from the MR CLEAN Registry [12], a registry of consecutive stroke patients treated with EVT in the Netherlands. An initial selection of pre- and post-EVT sequences from the MR CLEAN Registry is adopted from a previous study [4]. A subsequent selection is done to reduce annotation time while retaining a representative view of clinical variability. This resulted in 104 patients to be included, of which the pre-/post-EVT AP and lateral DSA series are evaluated. Figure 1 is one such patient record. During the U-net model training and validation, procedural recordings of other patients were used: 1716 AP and 1472 lateral series in total.

## Experiments and results

### Intra-patient manual transformation assessment

To assess the impact of additional degrees of freedom on alignment accuracy, global transformations are optimized for manually annotated pre- and post-EVT recordings of 104 patients. Image pairs with fewer than six point correspondences are excluded to prevent overfitting. The resulting error distributions per transformation type are shown in Fig. 2.

### Landmark detection

For the assessment of the U-net-based landmark detection, we performed a threefold cross-validation. In this cross-validation, the data are randomly split based on patient id, thereby preventing validation and training on images from the same patient. Models were trained using different loss functions and the Adam optimizer until convergence was achieved. Weights were saved for the epoch with the best centre-of-mass prediction error on the validation set. The results are shown in Fig. 3.

### Point-based registration

We examined transformations computed with automatically identified point-correspondences using the landmark model, SIFT, and ORB. The success rate of finding sufficient inliers ($\ge 5$) for combinations of these methods is summarised in Table 1.

**Table 1:** Number of solutions (TP+FP) and invalid solutions (FP) found using automatically identified point correspondences for 104 image pairs

Methods			AP		Lateral	
SIFT	ORB	LM	TP+FP	FP	TP+FP	FP
$\checkmark$	$\times$	$\times$	58	0	65	2
$\times$	$\checkmark$	$\times$	101	23	103	21
$\checkmark$	$\checkmark$	$\times$	**101**	**19**	**104**	**16**
$\checkmark$	$\times$	$\checkmark$	67	1	76	1
$\times$	$\checkmark$	$\checkmark$	102	23	104	19
$\checkmark$	$\checkmark$	$\checkmark$	101	20	103	16

Bold values indicate the solution with most successful registrations

Solutions with an average error greater than 10*px* are classified as invalid, based on Fig. 2

**Fig. 4:**
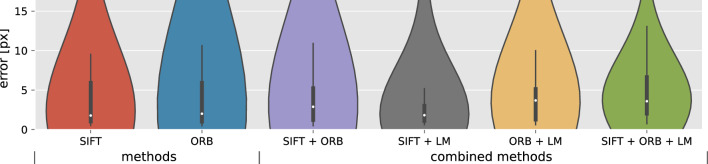
Registration error for least-squares similarity transformations using automatically identified point correspondences for lateral DSA images

Accuracy distributions of the methods, excluding the invalid solutions, are shown in Fig. 4. The range of the registration error is equivalent to Fig. 2. Each distribution represents a different image subset, i.e. the valid solutions of the method. For an unbiased method comparison, see Appendix B.7.

## Discussion and conclusion

We have investigated approaches to automatically align cerebral DSA series. Transformations that account for differences in scale are capable of aligning cerebral DSA sequences. Transformations with additional degrees of freedom are marginally more accurate. Although this could be attributed to improved modelling of projection of 3D motion, it is more likely a consequence of overfitting.

A deep-learning strategy using the U-net architecture proved capable of identifying cerebral artery landmarks to 4*px* accuracy. Performing image registration using the two landmarks proved limited, only yielding improved translation. Automatic image registration of pre-/post-EVT DSA sequences can, however, be performed using traditional point-based methods. SIFT produces negligible outliers with a lower success rate than ORB, which finds more solutions (+40%) at the cost of an increased false discovery rate (+20%). The accuracy of the point-based methods approaches the residual alignment error of manual annotations.

Combined, an 85% success rate is achieved with comparable performance for various types of stroke patients and procedural outcomes. This will enable further automation of DSA image analysis and procedure evaluation, contributing to outcome prediction and procedural decision-making for EVT.

### Supplementary Information

Below is the link to the electronic supplementary material.

Supplementary file 1 (pdf 186 KB)

Supplementary file 2 (pdf 1656 KB)
