# Supplementary material for: Automated image registration of cerebral digital subtraction angiography
Source: Int J Comput Assist Radiol Surg. 2023 Jul 17;19(1):147–50. doi: 10.1007/s11548-023-02999-8 (PMC10770205; doi:10.1007/s11548-023-02999-8)
Supplement: Supplementary file 1 — (pdf 186 KB) [file 11548_2023_2999_MOESM1_ESM.pdf]

A Least squares solutions for global transformations

A.1 Translation

For all linear global transformations, translation can be separately optimized:

$$\vec{x}_{target} = T\vec{x}_{source} + \vec{b}$$

$$\vec{x}'_{target} = \vec{x}_{target} - \bar{\vec{x}}_{target} \qquad \vec{x}'_{source} = \vec{x}_{source} - \bar{\vec{x}}_{source}$$

$$\min_{T,\vec{b}} \vec{x}_{target} - T\vec{x}_{source} - \vec{b}_2^2 =$$

$$\min_{T,\vec{b}} \vec{x}'_{target} + \bar{\vec{x}}_{target} - T\vec{x}_{source} - T\bar{\vec{x}}_{source} - \vec{b}_2^2 =$$

$$\min_{T} \vec{x}'_{target} - T\vec{x}'_{source 2} + \min_{T,\vec{b}} \bar{\vec{x}}_{target} - T\bar{\vec{x}}_{source} - \vec{b}_2^2$$

Where the second optimization problem is simply solved using the solution from the first optimization problem

$$\min_{T,\vec{b}} \bar{\vec{x}}_{target} - T\bar{\vec{x}}_{source} - \vec{b}_2^2$$

$$\vec{b} = \bar{\vec{x}}_{target} - T\bar{\vec{x}}_{source} \tag{1}$$

Which in the case of a pure translation becomes

$$\vec{b} = \bar{\vec{x}}_{target} - \bar{\vec{x}}_{source} \tag{2}$$

A.2 Rigid and Affine

$$\vec{x}'_{target} = T\vec{x}'_{source}$$

Let the source and target points compose a matrix

$$X'_{target} = [\vec{x}'_{target_1}, \vec{x}'_{target_2}, ...\vec{x}'_{target_N}] \quad X'_{source} = [\vec{x}'_{source_1}, \vec{x}'_{source_2}, ...\vec{x}'_{source_N}]$$

Then the least squares affine transformation is given by:

$$T = X'_{target}(X'_{source}X'^{T}_{source})^{-1} \tag{3}$$

and the rigid transformation, the closest orthogonal matrix with a positive determinant, is found using a singular value decomposition.

$$T = U\Sigma V^T$$

$$T_{rigid} = U \begin{bmatrix} 1 & 0 \\ 0 & sign(det(UV^T)) \end{bmatrix} V^T \tag{4}$$

Note that, since this is a 2×2 matrix (2D), or 3×3 (3D), an exact singular value decomposition can be computed.

A.3 Similarity

Rewriting the transformation into the following form provides a simple least-squares solution:

$$\begin{bmatrix} x'_{source_1} & -y'_{source_1} \\ y'_{source_1} & x'_{source_1} \\ x'_{source_2} & -y'_{source_2} \\ y'_{source_2} & x'_{source_2} \\ \vdots & \vdots \\ x'_{source_N} & -y'_{source_N} \\ y'_{source_N} & x'_{source_N} \end{bmatrix} \begin{bmatrix} s \times cos(\theta) \\ s \times sin(\theta) \end{bmatrix} = \begin{bmatrix} x'_{source_1} \\ y'_{source_1} \\ x'_{source_2} \\ y'_{source_2} \\ \vdots \\ x'_{source_N} \\ y'_{source_N} \end{bmatrix}$$

Having renamed the LHS matrix to X'_{source} and RHS matrix to X'_{target}, the solution becomes:

$$\begin{bmatrix} s \times cos(\theta) \\ s \times sin(\theta) \end{bmatrix} = (X'^{T}_{source} X'_{source})^{-1} X'^{T}_{source} X'_{target} \tag{5}$$

$$T_{similarity} = \begin{bmatrix} s \times cos(\theta) & -s \times sin(\theta) \\ s \times sin(\theta) & s \times cos(\theta) \end{bmatrix}$$

A.4 Projection

Direct linear transform (DLT)

$$x_{target} = \frac{a_{00}x_{source}+a_{01}y_{source}+a_{02}}{a_{20}x_{source}+a_{21}y_{source}+a_{20}} \quad y_{target} = \frac{a_{10}x_{source}+a_{11}y_{source}+a_{02}}{a_{20}x_{source}+a_{21}y_{source}+a_{20}} \tag{6}$$

Rewriting the equations will produce a linear system

$$(a_{20}x_{source} + a_{21}y_{source} + a_{22})x_{target} = a_{00}x_{source} + a_{01}y_{source} + a_{02}$$

$$(a_{20}x_{source} + a_{21}y_{source} + a_{22})y_{target} = a_{10}x_{source} + a_{11}y_{source} + a_{02}$$

and as long as the LHS matrix below is invertible, this will also provide an exact solution when using four points. When using more than four points, this does not provide a least squares solution to the original problem, but a good approximation:

$$\begin{bmatrix} x_{source_1} & y_{source_1} & 1 & 0 & 0 & 0 & x_{source_1}x_{target_1} & y_{source_1}x_{target_1} \\ 0 & 0 & 0 & x_{source_1} & y_{source_1} & 1 & x_{source_1}y_{target_1} & y_{source_1}y_{target_1} \\ x_{source_2} & y_{source_2} & 1 & 0 & 0 & 0 & x_{source_2}x_{target_2} & y_{source_2}x_{target_2} \\ 0 & 0 & 0 & x_{source_2} & y_{source_2} & 1 & x_{source_2}y_{target_2} & y_{source_2}y_{target_2} \\ \vdots & & & & & & & \\ x_{source_1} & y_{source_N} & 1 & 0 & 0 & 0 & x_{source_N}x_{target_N} & y_{source_N}x_{target_N} \\ 0 & 0 & 0 & x_{source_N} & y_{source_N} & 1 & x_{source_N}y_{target_N} & y_{source_N}y_{target_N} \end{bmatrix} \begin{bmatrix} a_{00} \\ a_{01} \\ a_{02} \\ a_{10} \\ a_{11} \\ a_{12} \\ a_{20} \\ a_{21} \end{bmatrix} = \begin{bmatrix} x_{source_1} \\ y_{source_1} \\ x_{source_2} \\ y_{source_2} \\ \vdots \\ x_{source_N} \\ y_{source_N} \end{bmatrix}$$

Renaming the LHS matrix to X, and the RHS vector to $\vec{x}$, the approximation of $\vec{a}$ becomes:

$$\vec{a} = (X^T X)^{-1} X^T \vec{x}$$

Simplification and exact computation of the DLT: The DLT solution uses an 8×8 matrix. One such implementation can be found in Open CV, which uses a numerical solver. An exact solution of an inverse matrix has complexity $\mathcal{O}(n!)$ (with $n = 8$). Firstly we can reduce the system to a 6×6 in a similar manner as Equation 1. Additionally, one can notice $(X^T X)^{-1}$ can be re-written in block form:

$$\begin{bmatrix} X_1 & 0_{2\times2} & X_2 \\ 0_{2\times2} & X_1 & X_3 \\ X_2^T & X_3^T & X_4 \end{bmatrix}^{-1} = \begin{bmatrix} X_1^{-1} + X_1^{-1} X_2 X_5^{-1} X_1^{-1} & X_1^{-1} X_2 X_5^{-1} X_2^T X_1^{-1} & -X_1^{-1} X_2 X_5^{-1} \\ X_1^{-1} X_3 X_5^{-1} X_2^T X_1^{-1} & X_1^{-1} + X_1^{-1} X_3 X_5^{-1} X_3^T X_1^{-1} & -X_1^{-1} X_3 X_5^{-1} \\ -X_5^{-1} X_2^T X_1^{-1} & -X_5^{-1} X_3^T X_1^{-1} & X_5^{-1} \end{bmatrix} \quad (7)$$

with

$$X_5^{-1} = (X_4 - X_2^T X_1^{-1} X_2 - -X_3^T X_1^{-1} X_3)^{-1}$$

such that the solution only requires two inverse matrices, both of size 2×2. In our application, one can be pre-computed. Furthermore, as we will elaborate next, we particularly want to know the values of a_{20} and a_{21}.

$$\begin{bmatrix} a_{20} \\ a_{21} \end{bmatrix} = \begin{bmatrix} -X_5^{-1} X_2^T X_1^{-1} & -X_5^{-1} X_3^T X_1^{-1} & X_5^{-1} \end{bmatrix} X^T \vec{x} \quad (8)$$

Exact solutions: While not all transformation parameters have a least-squares solution (a_{20} and a_{21}), most do. For completeness, the solutions are provided on the next page, although in practice it comes down to adapting X_{source} in Equation 3 such that the denominator of Equation 6 is included, with fixed values a_{20} and a_{21}.

Note that theoretically, there are many solutions for a_{20} and a_{21} as its solutions would be the roots of an excessively high-order bi-variate polynomial. Trying to solve this, and evaluating which of the solutions is the global optimum is complicated and numerical. Using the DLT for these two parameters is therefore the better alternative.

Additional constraints The denominator of Equation 6 should not be zero within the field of view (and in practice is never close by). The closest point of that line to the origin (i.e. the centre of the field of view) is:

$$\vec{x} = \begin{pmatrix} \frac{a_{20}}{a_{21}(1+\frac{a_{20}}{a_{21})}^2} \\ \frac{a_{20}^2}{(a_{20}+a_{21})^2} - \frac{1}{a_{21}} \end{pmatrix}$$

Its distance to the origin should therefore be constraint to prevent unstable behaviour; for example by enforcing $\vec{x}^T \vec{x} > \frac{h}{2}^2 + \frac{w}{2}^2$.

$$a_{02} = \alpha \sum_i x_{target_i} - \frac{a_{00}x_{source_i} + a_{01}y_{source_i}}{a_{20}x_{source_i} + a_{21}y_{source_i} + 1} \tag{9}$$

$$a_{12} = \alpha \sum_i y_{target_i} - \frac{a_{10}x_{source_i} + a_{11}y_{source_i}}{a_{20}x_{source_i} + a_{21}y_{source_i} + 1} \tag{10}$$

$$\begin{bmatrix} a_{00} & a_{10} \\ a_{01} & a_{11} \end{bmatrix} = Z^{-1} \begin{bmatrix} \sum_i x_{target_i}(x_{source_i} - \alpha\beta) & \sum_i y_{target_i}(x_{source_i} - \alpha\beta) \\ \sum_i x_{target_i}(y_{source_i} - \alpha\gamma) & \sum_i y_{target_i}(y_{source_i} - \alpha\gamma) \end{bmatrix} \tag{11}$$

$$\alpha = (\sum_j \frac{1}{a_{20}x_{source_i} + a_{21}y_{source_i} + 1})^{-1}$$

$$\beta = \sum_j \frac{x_{source_j}}{a_{20}x_{source_j} + a_{21}y_{source_j} + 1})$$

$$\gamma = \sum_j \frac{y_{source_j}}{a_{20}x_{source_j} + a_{21}y_{source_j} + 1})$$

$$Z = \begin{bmatrix} \sum_i \frac{x_{source_i}(x_{source_i} + \alpha\beta)}{a_{20}x_{source_i} + a_{21}y_{source_i} + 1} & \sum_i \frac{y_{source_i}(x_{source_i} + \alpha\beta)}{a_{20}x_{source_i} + a_{21}y_{source_i} + 1} \\ \sum_i \frac{x_{source_i}(y_{source_i} + \alpha\gamma)}{a_{20}x_{source_i} + a_{21}y_{source_i} + 1} & \sum_i \frac{y_{source_i}(y_{source_i} + \alpha\gamma)}{a_{20}x_{source_i} + a_{21}y_{source_i} + 1} \end{bmatrix}$$
