# Supplementary material for: Automated image registration of cerebral digital subtraction angiography
Source: Int J Comput Assist Radiol Surg. 2023 Jul 17;19(1):147–50. doi: 10.1007/s11548-023-02999-8 (PMC10770205; doi:10.1007/s11548-023-02999-8)
Supplement: Supplementary file 2 — (pdf 1656 KB) [file 11548_2023_2999_MOESM2_ESM.pdf]

B Supplementary data

B.1 Data pre-processing

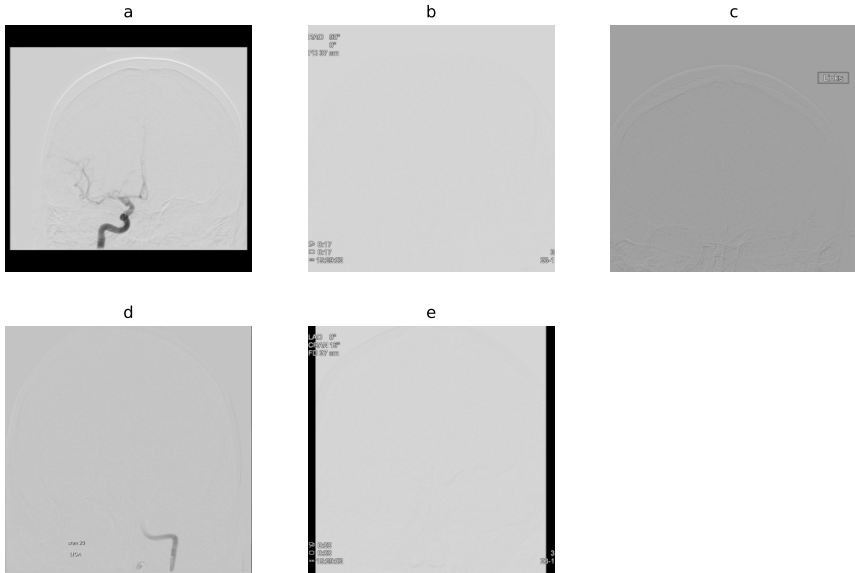

Fig. 1 Common artefacts in DSA sequences in the Mr Clean registry. a: border artefacts. b: embedded overlays. c: embedded text with bounding box (Allura Xper device) d: embedded text (Axiom artis device) e: Combinations of border artefacts and embedded overlays.

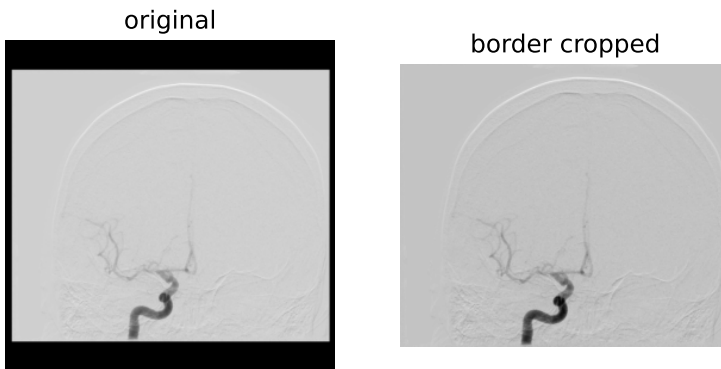

Fig. 2 Border removal. Border artefact in original image is automatically identified and cropped (or masked in the proceeding Figures).

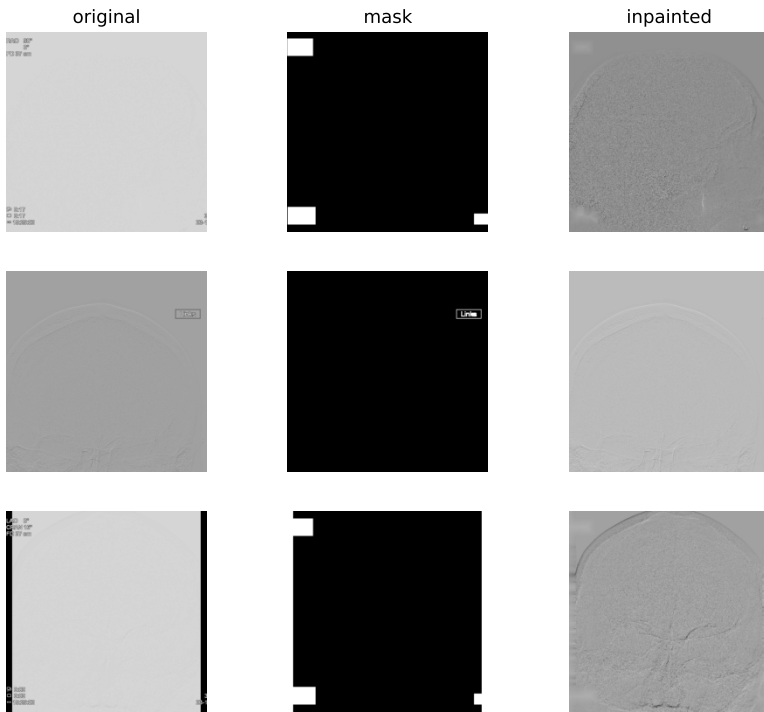

Fig. 3 Allura Xper pre-processing. The standard overlay and text with bounding box are automatically identified, masked and inpainted using Open CV.

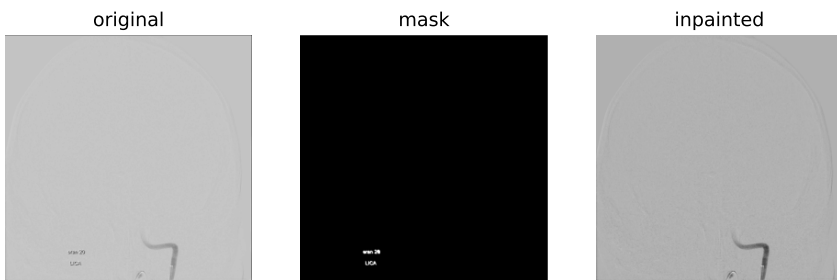

Fig. 4 Axiom Artis pre-processing. Text is automatically identified, masked and inpainted using Open CV. Borders are removed if identified.

B.2 Optimized transformations (lateral)

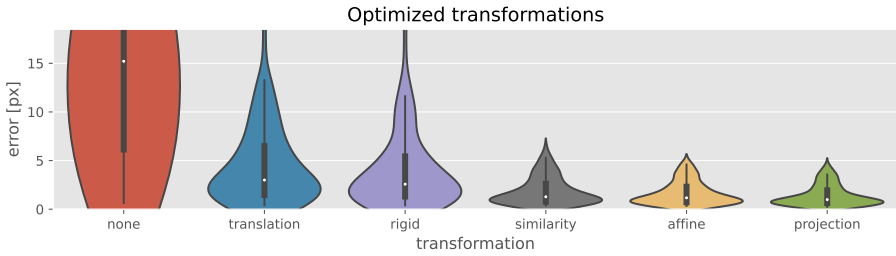

Fig. 5 Average alignment error for transformations computed using manual annotations from lateral MinIP pairs. The violin represents the data distribution and inside, the median (white), interquartile range (gray, thick) and inter-adjacent value range (gray, thin) are indicated.

B.3 Landmark model performance

The prediction error over the three fold validation for AP minIps. Two methods, argmax and centre of mass are used to infer the coordinate from the probability distributions.

Table 2 The prediction error over the three fold validation for AP minIps.

centre of mass average prediction error [<i>pixels</i>] (mean + s.d.)							
Loss	mean	25 th ICA	mean ICA	75 th ICA	25 th M1	mean M1	75 th M1
KL_{fw}	6.6 ± 0.7	1.9 ± 0.3	7.0 ± 0.7	6.9 ± 2.6	1.7 ± 0.3	6.2 ± 0.8	5.8 ± 1.7
KL_{bw}	5.9 ± 0.4	1.8 ± 0.3	6.1 ± 0.6	5.7 ± 1.6	1.6 ± 0.3	5.7 ± 0.4	5.1 ± 1.0
$KL_{fw} N$	5.8 ± 1.0	1.9 ± 0.5	5.6 ± 0.8	5.7 ± 1.2	2.1 ± 0.8	6.0 ± 1.3	6.6 ± 2.0
$KL_{bw} N$	6.4 ± 0.6	2.1 ± 0.5	5.9 ± 0.4	6.2 ± 1.5	2.9 ± 0.7	6.8 ± 0.8	7.6 ± 1.7
$KL_{fw} N_s$	8.5 ± 2.2	2.5 ± 0.4	8.0 ± 2.0	7.8 ± 1.7	3.5 ± 1.1	8.9 ± 2.4	10.0 ± 2.6
$KL_{bw} N_s$	9.9 ± 3.2	3.5 ± 1.0	9.0 ± 2.1	10.5 ± 2.5	5.6 ± 3.0	10.8 ± 4.3	13.8 ± 4.6
JS	6.4 ± 0.7	1.9 ± 0.4	6.9 ± 0.6	6.5 ± 2.0	1.5 ± 0.4	6.0 ± 0.8	5.5 ± 1.8
$p_{comb.1}$	**4.3 ± 0.4**	**1.6 ± 0.1**	**4.6 ± 0.3**	**4.4 ± 0.4**	**1.4 ± 0.2**	**4.1 ± 0.6**	**3.7 ± 0.3**
$p_{comb.2}$	4.9 ± 0.7	1.9 ± 0.3	4.9 ± 0.4	5.0 ± 0.6	1.9 ± 0.5	5.0 ± 1.0	5.7 ± 1.0
argmax prediction error [<i>pixels</i>] (mean + s.d.)							
Loss	mean	25 th ICA	mean ICA	75 th ICA	25 th M1	mean M1	75 th M1
KL_{fw}	5.1 ± 0.4	1.8 ± 0.3	5.3 ± 0.5	5.2 ± 1.4	1.5 ± 0.4	4.8 ± 0.5	4.4 ± 1.3
KL_{bw}	5.1 ± 0.4	1.8 ± 0.3	5.2 ± 0.5	4.9 ± 1.3	1.5 ± 0.4	4.9 ± 1.0	4.3 ± 0.9
$KL_{fw} N$	11.0 ± 1.2	7.2 ± 1.6	11.7 ± 1.6	13.0 ± 3.0	6.8 ± 0.7	10.4 ± 0.8	12.3 ± 1.5
$KL_{bw} N$	13.0 ± 1.8	9.1 ± 2.7	13.5 ± 1.5	16.2 ± 3.0	8.9 ± 2.5	12.6 ± 2.3	15.4 ± 4.1
$KL_{fw} N_s$	12.9 ± 7.2	5.7 ± 4.5	13.2 ± 7.3	13.0 ± 6.8	4.8 ± 2.4	12.5 ± 7.1	11.6 ± 4.1
$KL_{bw} N_s$	13.4 ± 1.5	5.0 ± 2.9	10.3 ± 2.7	11.6 ± 5.1	11.8 ± 4.4	16.4 ± 5.3	20.3 ± 4.8
JS	6.2 ± 0.2	1.8 ± 0.3	6.6 ± 0.8	5.5 ± 1.1	**1.3 ± 0.2**	5.9 ± 1.2	4.3 ± 1.2
$p_{comb.1}$	**4.6 ± 0.5**	**1.6 ± 0.3**	**5.0 ± 0.6**	**4.5 ± 0.4**	**1.3 ± 0.2**	**4.1 ± 0.5**	**3.8 ± 0.4**
$p_{comb.2}$	11.1 ± 1.4	6.9 ± 1.5	11.8 ± 1.9	12.6 ± 2.7	6.5 ± 0.5	10.4 ± 1.0	11.8 ± 1.0

B.4 Landmark model t-test comparisons

Table 3 The prediction error over the three fold validation for lateral minIps.

centre of mass average prediction error [<i>pixels</i>] (mean + s.d.)							
Loss	mean	25 th ICA	mean ICA	75 th ICA	25 th M1	mean M1	75 th M1
KL_{fw}	5.5 ± 0.9	1.6 ± 0.3	5.3 ± 0.9	4.3 ± 0.4	1.9 ± 0.5	5.7 ± 0.9	5.4 ± 1.0
KL_{bw}	5.3 ± 1.0	1.4 ± 0.2	5.3 ± 1.2	4.0 ± 0.4	1.9 ± 0.4	5.4 ± 0.8	5.0 ± 0.7
$KL_{fw} N$	4.8 ± 0.9	1.4 ± 0.2	4.7 ± 1.1	3.8 ± 0.3	1.8 ± 0.4	4.9 ± 0.7	4.9 ± 0.8
$KL_{bw} N$	5.7 ± 0.6	2.2 ± 0.4	5.6 ± 0.8	5.5 ± 0.7	2.3 ± 0.6	5.7 ± 0.6	6.5 ± 1.2
$KL_{fw} N_s$	10.7 ± 3.9	3.3 ± 0.2	10.8 ± 3.4	10.5 ± 2.2	3.5 ± 0.3	10.6 ± 4.4	11.3 ± 3.3
$KL_{bw} N_s$	8.6 ± 2.1	4.6 ± 3.0	9.0 ± 2.2	10.2 ± 4.0	3.9 ± 2.0	8.3 ± 2.1	10.1 ± 4.7
JS	5.2 ± 0.6	1.4 ± 0.3	4.7 ± 0.8	3.7 ± 0.6	1.8 ± 0.4	5.7 ± 0.5	5.2 ± 1.0
$p_{comb.1}$	**3.9 ± 0.4**	**1.3 ± 0.1**	**3.7 ± 0.5**	**3.3 ± 0.1**	**1.6 ± 0.3**	**4.1 ± 0.4**	**4.4 ± 0.2**
$p_{comb.2}$	4.2 ± 0.3	1.4 ± 0.2	4.1 ± 0.4	3.8 ± 0.2	1.7 ± 0.2	4.3 ± 0.3	4.7 ± 0.1

argmax prediction error [<i>pixels</i>] (mean + s.d.)							
Loss	mean	25 th ICA	mean ICA	75 th ICA	25 th M1	mean M1	75 th M1
KL_{fw}	4.3 ± 0.7	1.3 ± 0.2	3.8 ± 0.9	3.4 ± 0.3	**1.7 ± 0.5**	4.8 ± 0.8	4.9 ± 0.7
KL_{bw}	4.6 ± 0.6	1.3 ± 0.2	4.3 ± 0.6	3.5 ± 0.5	1.8 ± 0.6	5.0 ± 0.6	4.7 ± 0.8
$KL_{fw} N$	10.2 ± 0.6	7.8 ± 0.3	10.6 ± 1.3	11.5 ± 0.1	7.0 ± 0.5	9.8 ± 0.0	11.9 ± 0.8
$KL_{bw} N$	11.3 ± 0.2	9.6 ± 1.9	12.9 ± 1.2	15.5 ± 1.6	6.7 ± 1.8	9.7 ± 1.2	12.1 ± 2.4
$KL_{fw} N_s$	26.6 ± 9.5	10.5 ± 2.2	25.2 ± 6.5	24.6 ± 5.4	9.7 ± 2.2	28.0 ± 12.5	30.1 ± 15.2
$KL_{bw} N_s$	10.5 ± 2.8	6.3 ± 3.6	11.8 ± 2.8	14.1 ± 5.9	3.9 ± 2.4	9.2 ± 2.9	11.1 ± 5.1
JS	4.9 ± 1.1	1.3 ± 0.2	4.3 ± 1.1	3.5 ± 0.5	**1.7 ± 0.5**	5.5 ± 1.2	4.9 ± 1.0
$p_{comb.1}$	**3.9 ± 0.4**	**1.1 ± 0.2**	**3.6 ± 0.5**	**3.2 ± 0.0**	1.8 ± 0.3	**4.2 ± 0.5**	**4.3 ± 0.1**
$p_{comb.2}$	10.0 ± 0.3	7.8 ± 0.3	10.4 ± 0.7	11.4 ± 0.6	6.8 ± 0.5	9.7 ± 0.8	11.6 ± 0.8

Table 4 Students t-test comparing the model performance trained with different loss functions. H_0 : the average argmax prediction error is smaller for *model*₁ than for *model*₂. Probabilities are computed over the three fold validation. In bold if more than 95% confidence.

<i>model</i> ₁ \ <i>model</i> ₂	KL_{fw}	KL_{bw}	$KL_{fw} N$	$KL_{bw} N$	$KL_{fw} N_s$	$KL_{bw} N_s$	JS	$p_{comb.1}$	$p_{comb.2}$
KL_{fw}		0.15	0.21	0.36	0.84	0.88	0.41	0.01	0.04
KL_{bw}	0.85		0.45	0.79	0.91	0.92	0.81	0.01	0.08
$KL_{fw} N$	0.79	0.55		0.73	0.9	0.92	0.75	0.07	0.19
$KL_{bw} N$	0.64	0.21	0.27		0.87	0.9	0.55	0.01	0.05
$KL_{fw} N_s$	0.16	0.09	0.1	0.13		0.69	0.14	0.03	0.05
$KL_{bw} N_s$	0.12	0.08	0.08	0.1	0.31		0.11	0.04	0.05
JS	0.59	0.19	0.25	0.45	0.86	0.89		0.01	0.04
$p_{comb.1}$	**0.99**	**0.99**	0.93	**0.99**	**0.97**	**0.96**	**0.99**		0.82
$p_{comb.2}$	**0.96**	0.92	0.81	**0.95**	**0.95**	**0.95**	**0.96**	0.18	

Table 5 Students t-test comparing the lateral model performance trained with different loss functions. H_0 : the average argmax prediction error is smaller for *model*₁ than for *model*₂. Probabilities are computed over the three fold validation. In bold if more than 95% confidence.

<i>model</i> ₁ \ <i>model</i> ₂	KL_{fw}	KL_{bw}	$KL_{fw} N$	$KL_{bw} N$	$KL_{fw} N_s$	$KL_{bw} N_s$	JS	$p_{comb.1}$	$p_{comb.2}$
KL_{fw}		0.43	0.23	0.58	0.93	0.94	0.36	0.04	0.06
KL_{bw}	0.57		0.3	0.65	0.94	0.94	0.45	0.07	0.1
$KL_{fw} N$	0.77	0.7		0.84	0.95	**0.96**	0.69	0.13	0.21
$KL_{bw} N$	0.42	0.35	0.16		0.93	0.93	0.25	0.01	0.02
$KL_{fw} N_s$	0.07	0.06	0.05	0.07		0.27	0.06	0.03	0.04
$KL_{bw} N_s$	0.06	0.06	0.04	0.07	0.73		0.05	0.02	0.02
JS	0.64	0.55	0.31	0.75	0.94	**0.95**		0.03	0.06
$p_{comb.1}$	**0.96**	0.93	0.87	**0.99**	**0.97**	**0.98**	**0.97**		0.78
$p_{comb.2}$	0.94	0.9	0.79	**0.98**	**0.96**	**0.98**	0.94	0.22	

B.5 Landmark model training curves and metrics

The implicit loss functions display unstable behaviour. An attempt was made to improve stability by incorporating a smoothing kernel $\sigma = 0.5$ before computing the loss. This had a negative effect and was not resolved.

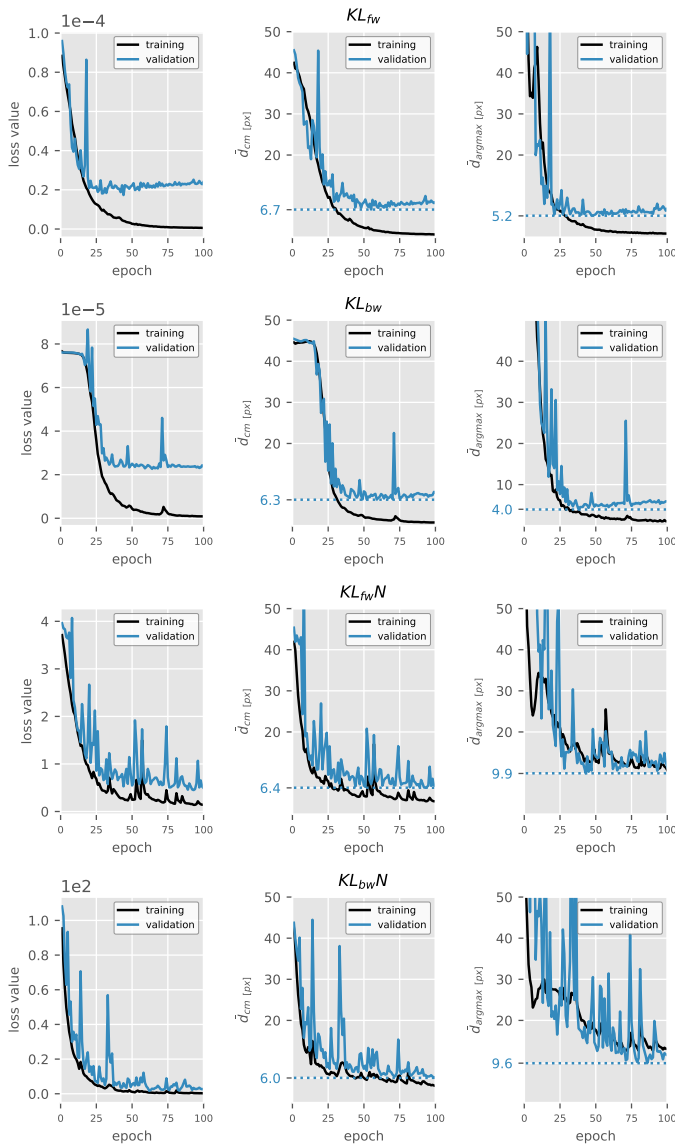

Fig. 6 Loss curves (first column), centre-of-mass error (second column) and argmax error (third column) for loss functions indicated in the centre column and trained on AP images

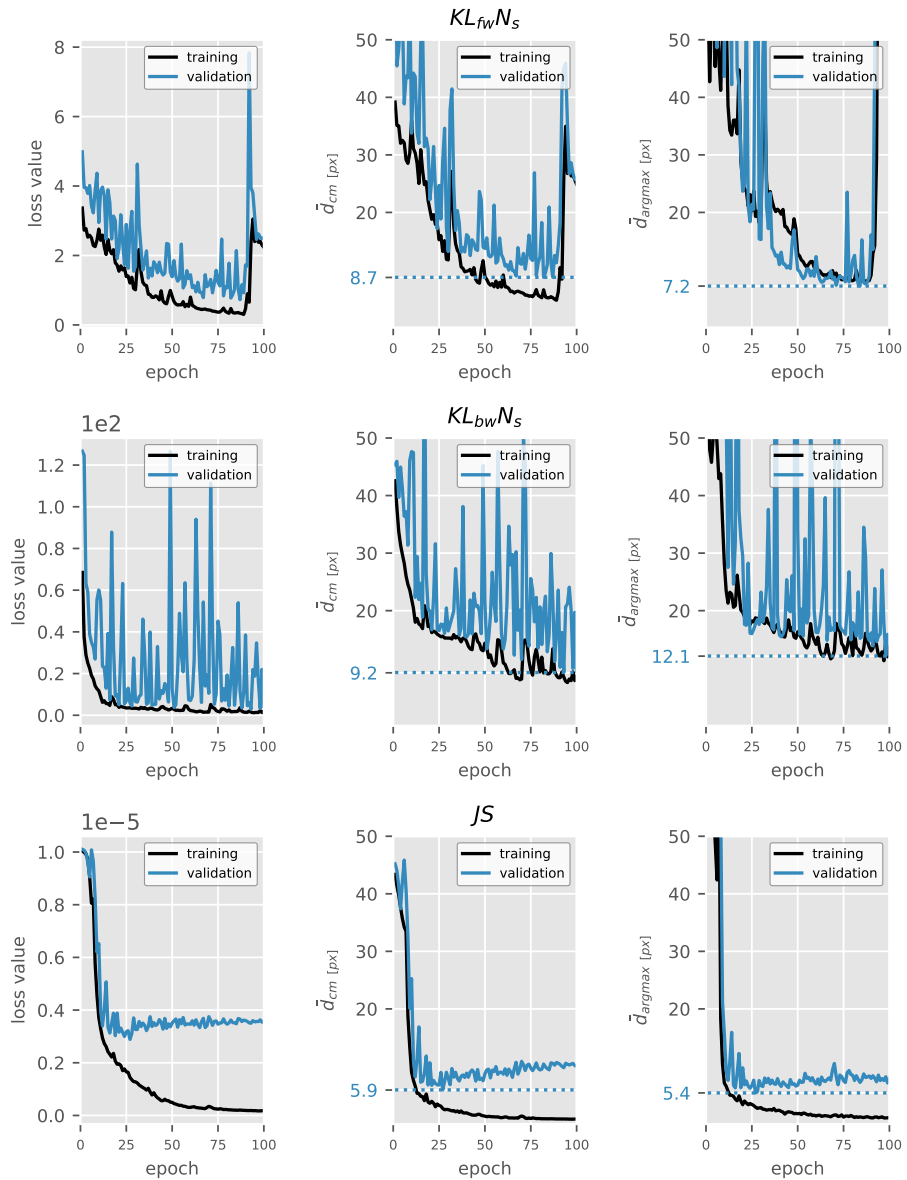

Fig. 7 Loss curves (first column), centre-of-mass error (second column) and argmax error (third column) for loss functions indicated in the centre column and trained on AP images

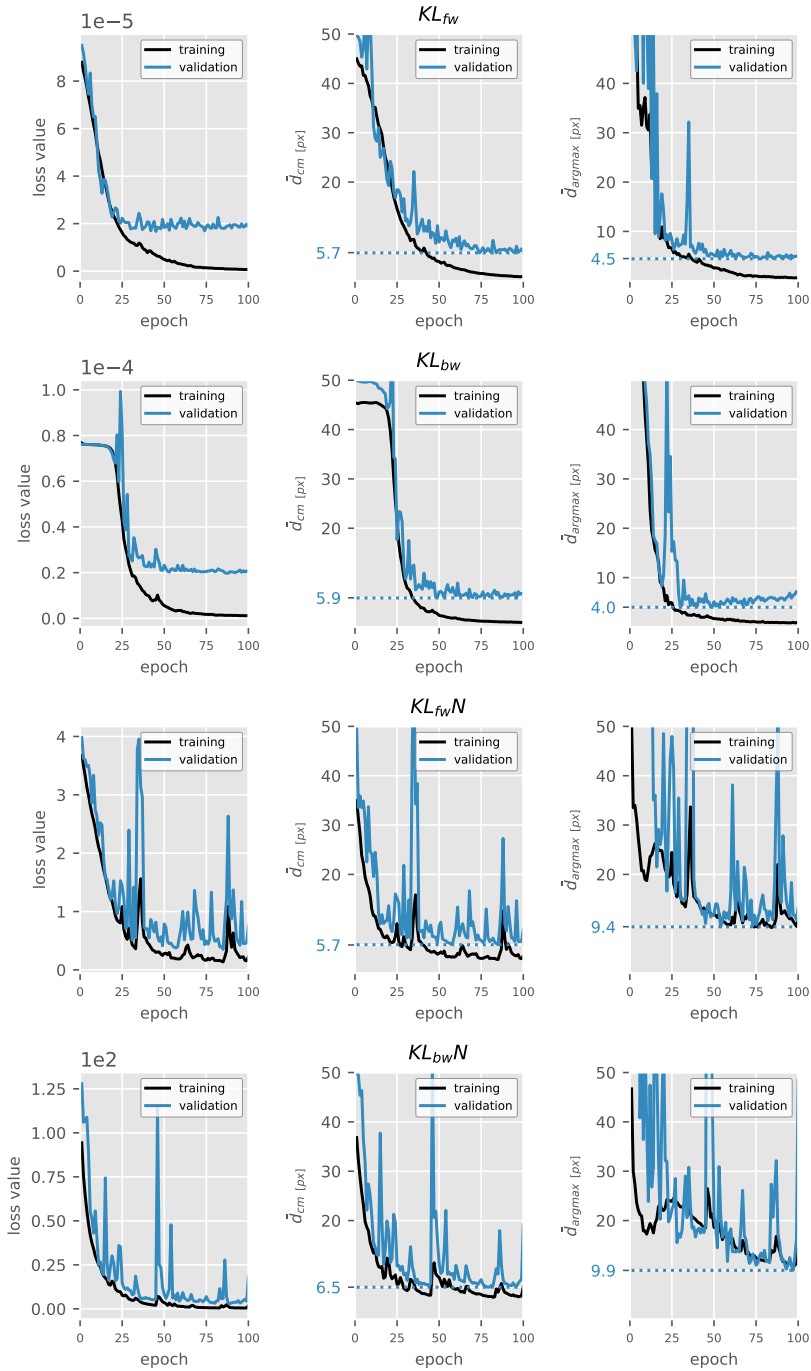

Fig. 8 Loss curves (first column), centre-of-mass error (second column) and argmax error (third column) for loss functions indicated in the centre column and trained on lateral images

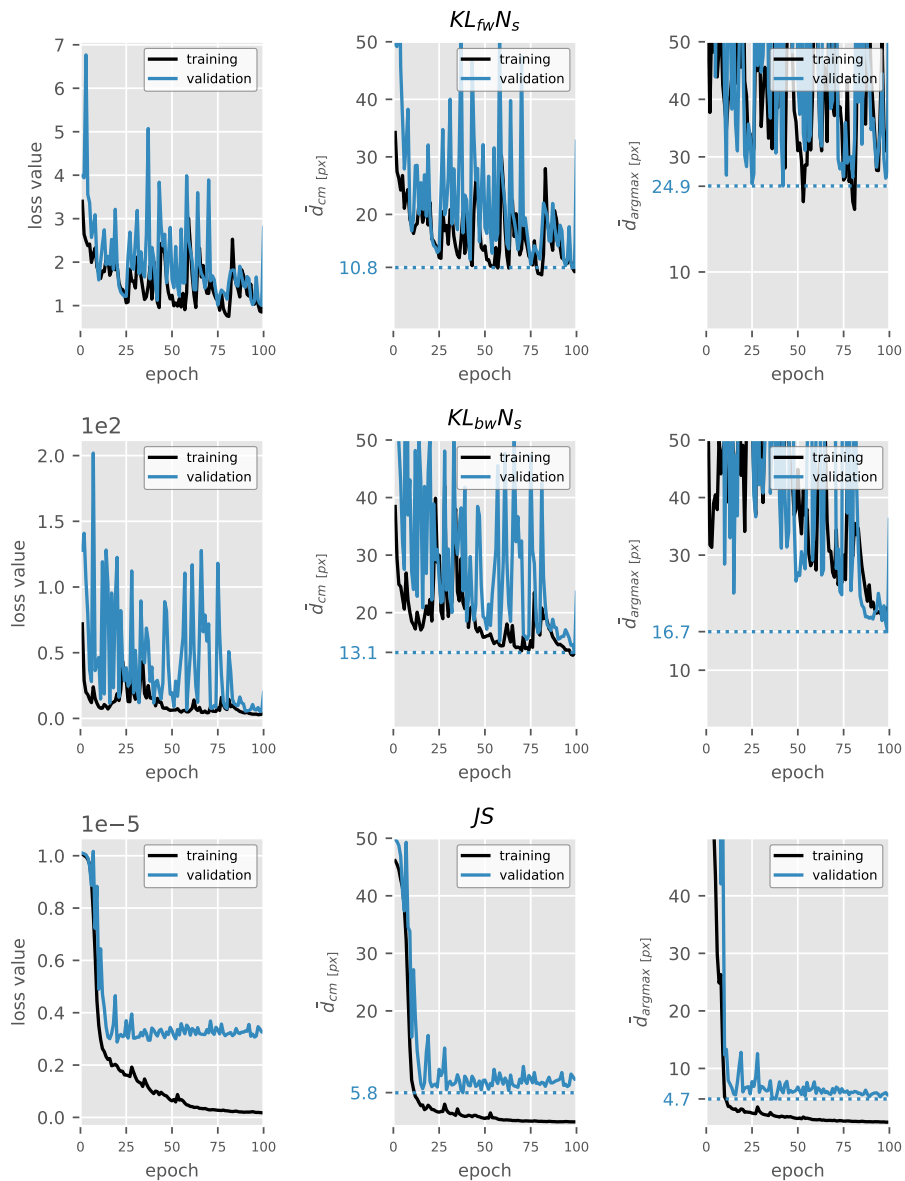

Fig. 9 Loss curves (first column), centre-of-mass error (second column) and argmax error (third column) for loss functions indicated in the centre column and trained on lateral images

B.6 Landmark-based registration

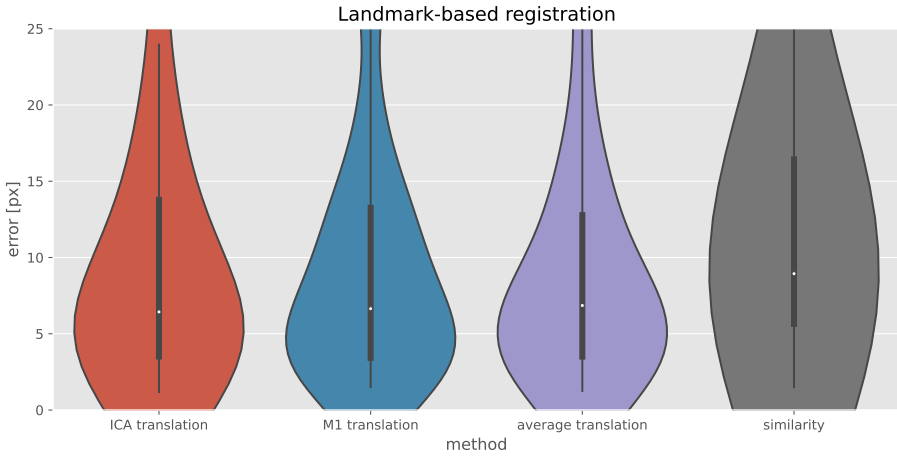

Fig. 10 Registration error using the only the landmark point correspondences for AP DSA MinIPs.

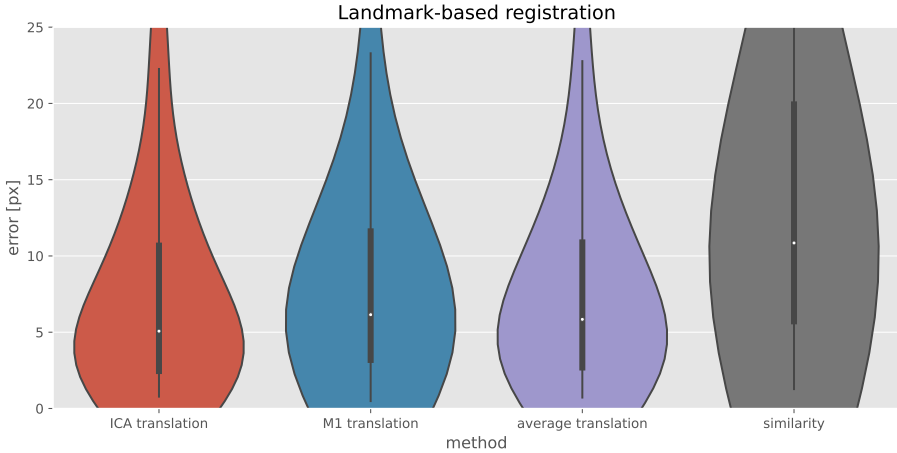

Fig. 11 Registration error using the only the landmark point correspondences for lateral DSA MinIPs.

W. Hellebrekers et al.

Table 1. Z-test comparing different methods, norms and transformations for AP images. The probability that the two methods are equivalent. If method (row) has a worse mean, the probability is set to one. The best performing method is highlighted in bold.

[illegible]

Table 2. Z-test comparing different methods, norms and transformations on Lateral images. The probability that the two methods are equivalent. If method (row) has a worse mean, the probability is set to one. The best performing method is highlighted in bold.

Method	Transformation	SIFT			ORB			SIFT			SIFT			ORB LM			ORB LM			Projection			Projection														
		Similarity	Affine	LM												
SIFT	norm	1.00	1.00	0.47	0.48	0.02	0.02	0.36	0.65	0.08	0.18	0.00	0.00	1.00	1.00	1.00	0.11	0.13	0.00	1.00	1.00	0.62	0.61	0.01	0.01	0.43	0.78	0.10	0.32	0.00	1.00	1.00	1.00	0.07	0.09		
	Similarity	1.00	0.79	1.00	0.33	0.34	0.00	0.00	0.00	0.00	0.00	0.00	0.00	0.00	0.00	0.00	0.00	0.00	0.00	0.00	0.00	0.00	0.00	0.00	0.00	0.00	0.00	0.00	0.00	0.00	0.00	0.00	0.00	0.00	0.00		
	Affine	1.00	1.00	1.00	0.99	1.00	0.02	0.02	0.89	1.00	0.35	0.50	0.01	0.01	1.00	1.00	1.00	0.32	0.37	1.00	1.00	1.00	1.00	0.01	0.01	1.00	1.00	1.00	0.84	0.81	0.01	1.00	1.00	1.00	0.21	0.26	
	Projection	1.00	1.00	1.00	1.00	1.00	1.00	1.00	1.00	1.00	1.00	1.00	1.00	1.00	1.00	1.00	1.00	1.00	1.00	1.00	1.00	1.00	1.00	1.00	1.00	1.00	1.00	1.00	1.00	1.00	1.00	1.00	1.00	1.00	1.00		
	Similarity	1.00	1.00	1.00	1.00	1.00	1.00	1.00	1.00	1.00	1.00	1.00	1.00	1.00	1.00	1.00	1.00	1.00	1.00	1.00	1.00	1.00	1.00	1.00	1.00	1.00	1.00	1.00	1.00	1.00	1.00	1.00	1.00	1.00	1.00		
	Affine	1.00	1.00	0.77	0.78	0.02	0.02	0.98	1.00	0.86	0.88	0.18	0.05	1.00	1.00	1.00	0.32	0.35	1.00	1.00	0.89	0.88	0.00	0.00	1.00	1.00	1.00	0.29	0.19	1.00	1.00	1.00	0.74	0.61			
	Projection	1.00	1.00	1.00	1.00	1.00	1.00	1.00	1.00	1.00	1.00	1.00	1.00	1.00	1.00	1.00	1.00	0.32	0.38	1.00	1.00	0.32	0.38	0.01	0.01	1.00	1.00	1.00	0.30	0.21	1.00	1.00	1.00	0.88	0.72		
	Similarity	1.00	1.00	1.00	1.00	1.00	1.00	1.00	1.00	1.00	1.00	1.00	1.00	1.00	1.00	1.00	1.00	0.75	1.00	1.00	1.00	1.00	1.00	1.00	1.00	1.00	1.00	1.00	1.00	1.00	1.00	1.00	1.00	1.00	1.00		
	Affine	1.00	1.00	1.00	1.00	1.00	1.00	1.00	1.00	1.00	1.00	1.00	1.00	1.00	1.00	1.00	1.00	0.44	1.00	1.00	1.00	1.00	1.00	1.00	1.00	1.00	1.00	1.00	1.00	1.00	1.00	1.00	1.00	1.00	1.00		
	Projection	1.00	1.00	1.00	1.00	1.00	1.00	1.00	1.00	1.00	1.00	1.00	1.00	1.00	1.00	1.00	1.00	0.44	1.00	1.00	1.00	1.00	1.00	1.00	1.00	1.00	1.00	1.00	1.00	1.00	1.00	1.00	1.00	1.00	1.00		
	Similarity	1.00	0.34	0.75	0.18	0.28	0.02	0.02	0.27	0.29	0.20	0.21	0.16	0.03	0.97	1.00	0.89	0.88	0.30	0.22	0.85	0.73	0.13	0.10	0.00	0.01	0.51	0.34	0.03	0.53	0.26	0.10	0.98	0.93	0.26	0.21	
	SIFT ORB	norm	1.00	0.32	0.38	0.09	0.10	0.02	0.02	0.26	0.27	0.19	0.20	0.10	0.03	0.97	1.00	0.89	0.88	0.30	0.22	0.85	0.73	0.13	0.10	0.00	0.01	0.51	0.34	0.03	0.53	0.26	0.10	0.83	0.97	0.06	0.10
Similarity		1.00	0.52	0.72	0.18	0.19	0.02	0.02	0.32	0.33	0.23	0.24	0.16	0.03	1.00	1.00	1.00	1.00	0.31	0.23	1.00	1.00	0.51	0.50	0.00	0.00	0.60	0.64	0.36	0.40	0.27	0.11	1.00	1.00	0.23	0.22	
Affine		1.00	1.00	1.00	1.00	1.00	1.00	1.00	1.00	1.00	1.00	1.00	1.00	1.00	1.00	1.00	1.00	1.00	1.00	1.00	1.00	1.00	1.00	1.00	1.00	1.00	1.00	1.00	1.00	1.00	1.00	1.00	1.00	1.00	1.00		
Projection		1.00	1.00	1.00	1.00	1.00	1.00	1.00	1.00	1.00	1.00	1.00	1.00	1.00	1.00	1.00	1.00	1.00	1.00	1.00	1.00	1.00	1.00	1.00	1.00	1.00	1.00	1.00	1.00	1.00	1.00	1.00	1.00	1.00	1.00		
Similarity		1.00	0.92	1.00	0.40	0.41	0.02	0.02	0.21	0.29	0.04	0.07	0.05	0.06	1.00	1.00	0.75	0.79	0.02	0.03	1.00	1.00	0.31	0.31	0.00	0.00	0.27	0.53	0.04	0.11	0.09	0.16	0.85	1.00	0.03	0.03	
Affine		1.00	1.00	0.78	0.80	0.02	0.02	0.85	1.00	0.26	0.40	0.07	0.08	1.00	1.00	1.00	0.06	0.07	0.00	0.00	1.00	1.00	0.99	0.90	0.00	0.00	0.97	0.26	0.52	0.11	0.18	1.00	1.00	0.10	0.10		
Projection		1.00	1.00	1.00	1.00	1.00	1.00	1.00	1.00	1.00	1.00	1.00	1.00	1.00	1.00	1.00	1.00	1.00	1.00	1.00	1.00	1.00	1.00	1.00	1.00	1.00	1.00	1.00	1.00	1.00	1.00	1.00	1.00	1.00	1.00		
Similarity		1.00	1.00	1.00	1.00	1.00	1.00	1.00	1.00	1.00	1.00	1.00	1.00	1.00	1.00	1.00	1.00	1.00	1.00	1.00	1.00	1.00	1.00	1.00	1.00	1.00	1.00	1.00	1.00	1.00	1.00	1.00	1.00	1.00	1.00		
Affine		1.00	1.00	1.00	0.64	0.66	0.02	0.02	0.53	0.55	0.41	0.43	0.17	0.04	1.00	1.00	1.00	0.31	0.27	1.00	1.00	0.71	0.70	0.00	0.00	0.96	1.00	0.65	0.70	0.28	0.14	1.00	1.00	0.40	0.35		
Projection		1.00	1.00	1.00	1.00	1.00	1.00	1.00	1.00	1.00	1.00	1.00	1.00	1.00	1.00	1.00	1.00	0.32	0.32	1.00	1.00	0.32	0.31	0.00	0.01	1.00	1.00	1.00	0.29	0.17	1.00	1.00	1.00	0.60	0.55		
Similarity		1.00	1.00	1.00	1.00	1.00	1.00	1.00	1.00	1.00	1.00	1.00	1.00	1.00	1.00	1.00	1.00	0.65	1.00	1.00	1.00	1.00	1.00	1.00	1.00	1.00	1.00	1.00	1.00	1.00	1.00	1.00	1.00	1.00	1.00		
Affine		1.00	0.76	0.98	0.29	0.31	0.02	0.02	0.28	0.30	0.21	0.22	0.16	0.03	1.00	1.00	0.97	0.98	0.31	0.23	0.94	0.92	0.29	0.29	0.00	0.00	0.55	0.59	0.33	0.37	0.27	0.11	1.00	1.00	0.96	0.97	0.21
Projection	1.00	1.00	1.00	1.00	1.00	1.00	1.00	1.00	1.00	1.00	1.00	1.00	1.00	1.00	1.00	1.00	0.38	1.00	1.00	1.00	1.00	1.00	1.00	1.00	1.00	1.00	1.00	1.00	1.00	1.00	1.00	1.00	1.00	1.00			
Similarity	1.00	0.43	0.64	0.15	0.16	0.02	0.02	0.27	0.28	0.19	0.21	0.16	0.03	1.00	1.00	0.93	0.92	0.31	0.23	0.67	0.67	0.17	0.17	0.00	0.00	0.58	0.62	0.35	0.39	0.27	0.11	1.00	1.00	0.92	0.93	0.22	
Affine	1.00	1.00	1.00	1.00	1.00	1.00	1.00	1.00	1.00	1.00	1.00	1.00	1.00	1.00	1.00	1.00	0.22	1.00	1.00	1.00	1.00	1.00	1.00	1.00	1.00	1.00	1.00	1.00	1.00	1.00	1.00	1.00	1.00	1.00			
Projection	1.00	1.00	1.00	1.00	1.00	1.00	1.00	1.00	1.00	1.00	1.00	1.00	1.00	1.00	1.00	1.00	0.33	0.43	1.00	1.00	0.33	0.43	1.00	1.00	1.00	1.00	1.00	1.00	1.00	1.00	1.00	1.00	1.00	1.00	1.00		

B.8 Point-based registration violin plot (AP)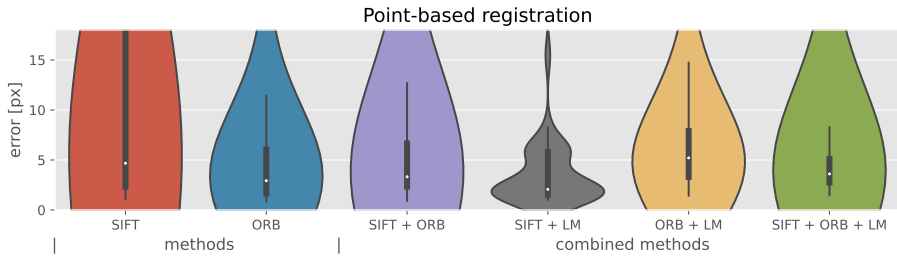

Fig. 12 Registration error (averaged distance between annotated point-correspondences) for least-squares similarity transformations using different subsets of automatically identified point correspondences in AP images.

B.9 Elastix registration Z-tests

Table 3: z-test for elastix method on AP images. If method (row) has a worse mean, the probability is set to one. The best performing elastix method is highlighted in bold.

Metric	MMI			MSD			NCC			SIFT ORB		
	Transformation	similarity	affine	similarity (initialized)	affine (initialized)	similarity (initialized)	similarity	affine	similarity (initialized)	similarity	affine (initialized)	similarity
MMI	similarity	1,00	0,03	0,56	0,58	0,01	0,29	1,00	1,00	0,72	0,02	1,00
	affine	1,00	1,00	1,00	1,00	0,92	1,00	1,00	1,00	1,00	0,39	1,00
	similarity (initialized)	1,00	0,08	1,00	1,00	0,05	0,62	1,00	0,06	1,00	0,04	1,00
MSD	affine	1,00	0,08	0,97	1,00	0,04	0,59	1,00	0,04	1,00	0,98	1,00
	similarity (initialized)	1,00	1,00	1,00	1,00	1,00	1,00	1,00	0,99	1,00	0,42	1,00
	affine	1,00	0,19	1,00	1,00	0,13	1,00	1,00	0,14	1,00	0,07	1,00
NCC	similarity (initialized)	0,56	0,01	0,28	0,28	0,01	0,13	1,00	0,01	1,00	0,48	0,01
	affine	1,00	1,00	1,00	1,00	1,00	1,00	1,00	1,00	1,00	0,50	1,00
	similarity (initialized)	1,00	0,01	0,21	0,21	0,00	0,09	0,89	0,01	1,00	0,42	0,01
SIFT ORB	affine	1,00	0,18	1,00	1,00	0,14	0,74	1,00	0,17	1,00	0,07	1,00
	similarity (initialized)	1,00	1,00	1,00	1,00	1,00	1,00	1,00	1,00	1,00	1,00	1,00
	affine	1,00	0,04	0,63	0,65	0,02	0,36	1,00	0,03	1,00	0,75	1,00
	similarity	0,33	0,01	0,14	0,17	0,00	0,05	0,75	0,01	0,88	0,39	0,01

B.10 Elastix registration violin plot

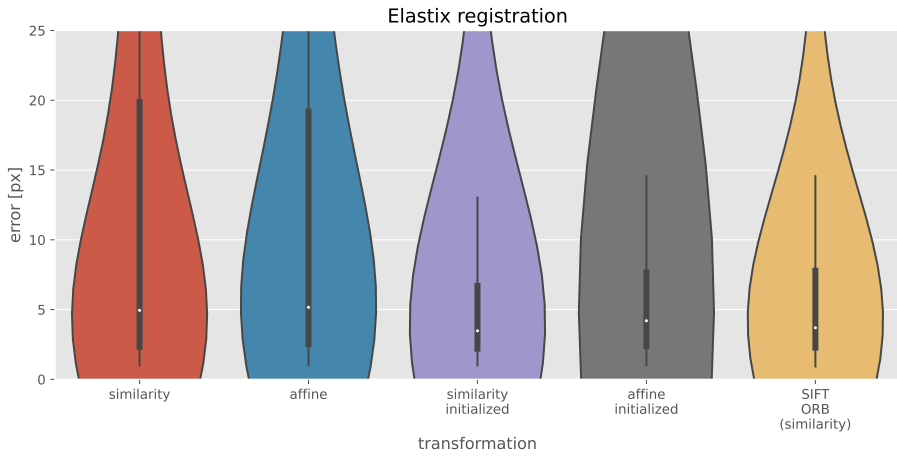

Fig. 13 Results of elastix registration optimizing mattes mutual information for AP images

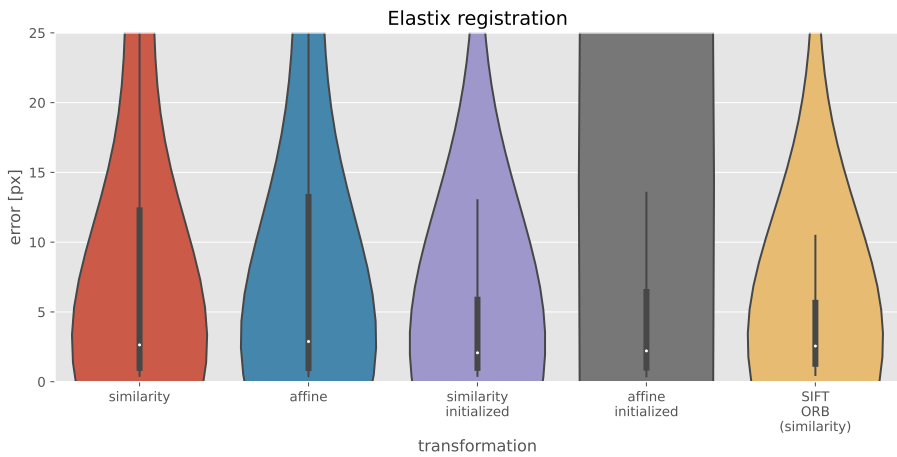

Fig. 14 Results of registration optimizing mattes mutual information for lateral images.

B.11 Automatic registration examples

Images are aligned using SIFT and ORB points to compute the similarity transformation.

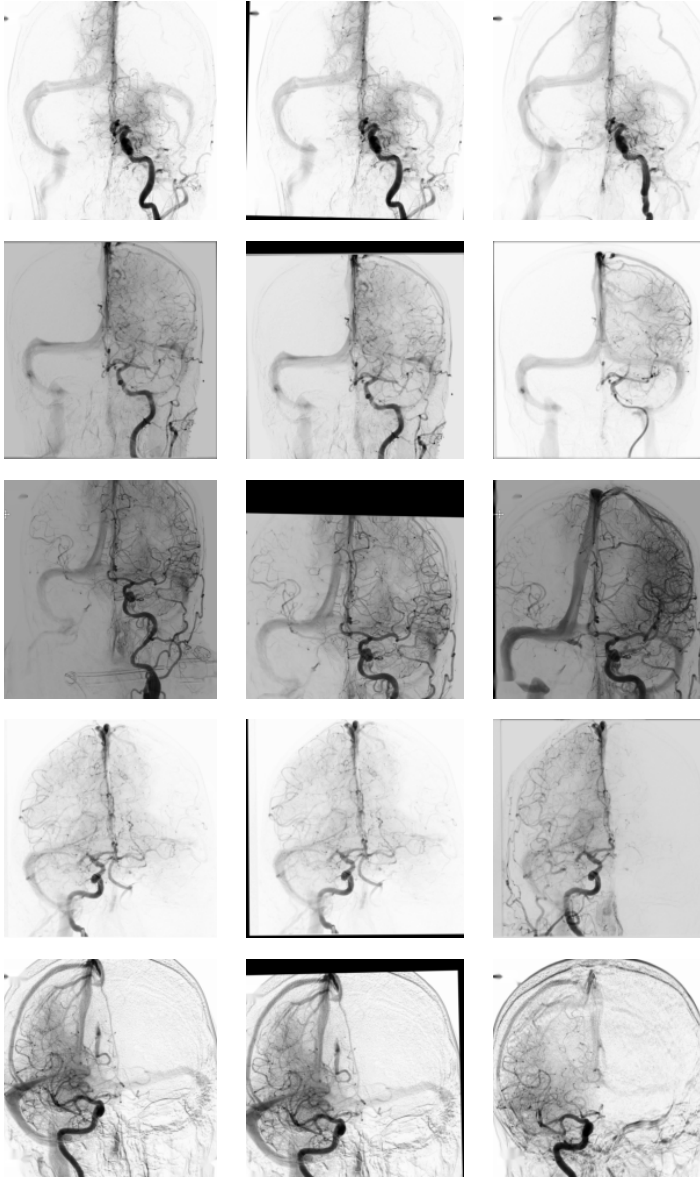

Fig. 15 AP registration examples: left column pre-EVT, middle is pre-EVT aligned to post-EVT in the right column.

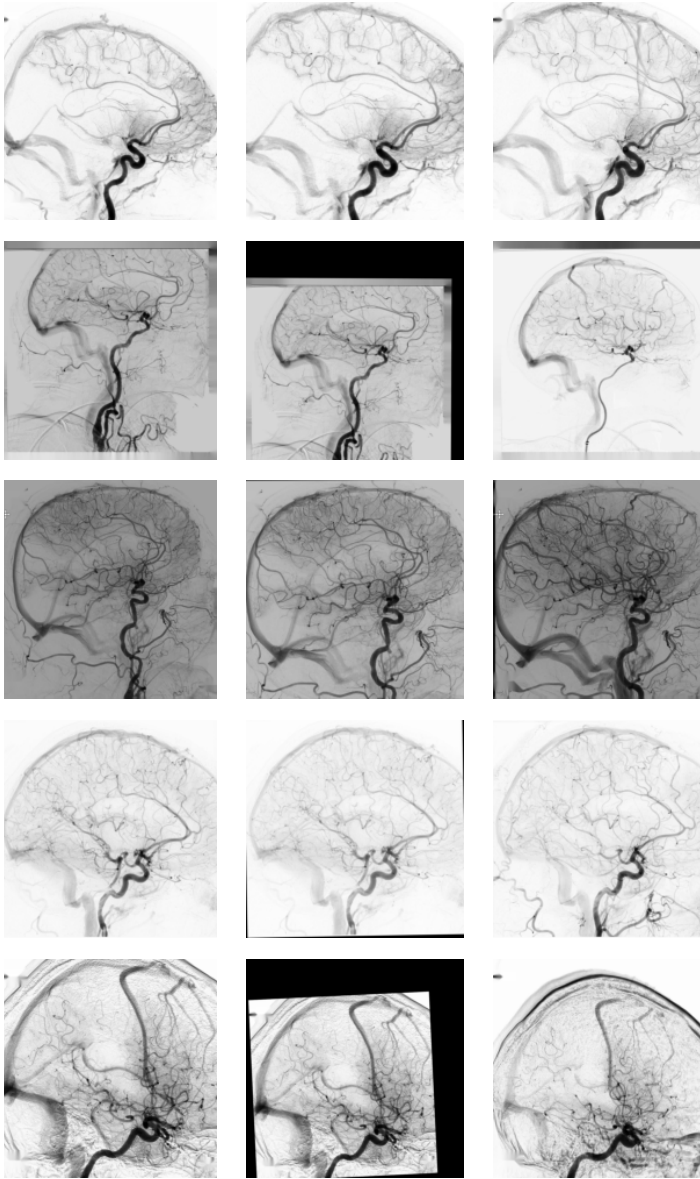

Fig. 16 Lateral registration examples: left column pre-EVT, middle is pre-EVT aligned to post-EVT in the right column.
